# Open Dorsal Proximal Interphalangeal Dislocation

**DOI:** 10.5811/cpcem.2019.12.45026

**Published:** 2020-04-14

**Authors:** Ryan Derrah, Cameron Wolterstorff

**Affiliations:** Madigan Army Medical Center, Department of Emergency Medicine, Tacoma, Washington

## Abstract

We report a case of a 44-year-old male with an uncommon case of an open dorsal proximal interphalangeal (PIP) dislocation. Although open PIP dislocations are often volar, dorsal dislocations are fraught with complications due to the potential for infection and damage to supportive structures. Features of this case are discussed together with its implications, including lack of standardized management in the literature, use of a closed reduction following copious irrigation, and requirement for antibiotic use.

## INTRODUCTION

Dislocations to the proximal interphalangeal (PIP) joint are common in athletes and are typically dorsal, resulting from an axial load on a hyperextended digit.^1^ Open dislocations are more commonly seen with volar dislocations and less commonly with dorsal ones.^2^ Open injuries may not respond to normal manual reduction techniques, and therefore require surgical, open reduction.^2^,^3^,^4^,^5^

## CASE REPORT

**Image 1:**
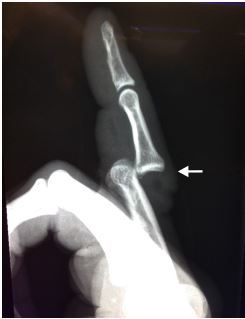
Lateral radiograph of right index finger demonstrating dorsal dislocation of the proximal interphalangeal joint without evidence of fracture (arrow).

A 44-year-old man, right-hand dominant, presented to our emergency department with pain and deformity to his right index finger. The injury had occurred just prior to arrival when his finger was struck while playing basketball. Radiographs from triage revealed a dorsal dislocation of the second PIP joint without evidence of fracture (Image 1).

**Image 2:**
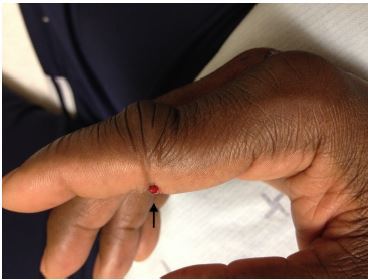
Dorsal displacement and deformity to right index finger with laceration to volar portion of the finger (arrow).

**Image 3:**
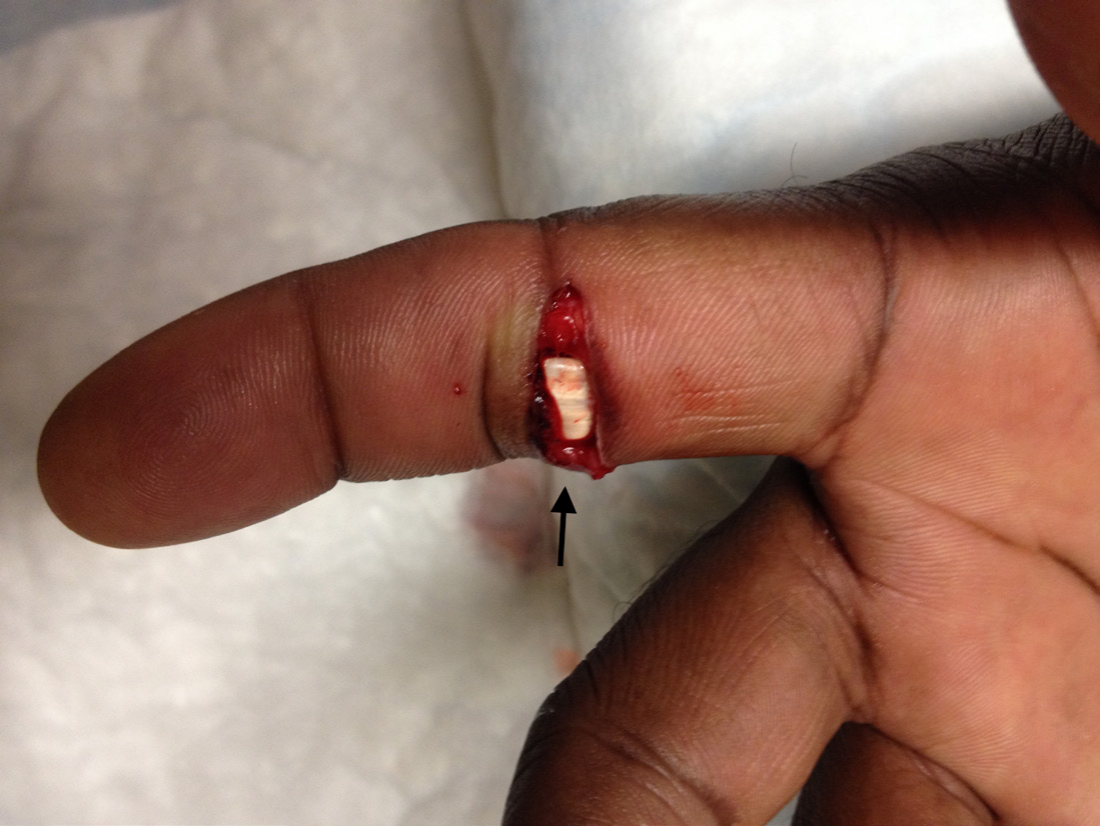
Laceration over volar proximal interphalangeal joint, exposing flexor tendon (arrow).

On exam, the middle phalanx was displaced dorsally and there was a transverse laceration to the volar surface of the PIP joint exposing the flexor tendon (Images 2 and 3). This was consistent with an open PIP dislocation. Since the patient was neurovascularly intact and orthopedics was readily available, immediate reduction was not attempted and the consulting service contacted. Orthopedics evaluated the patient, anesthetized the digit, copiously irrigated the wound, reduced the dislocation, sutured, and applied a splint. Orthopedics did not recommend antibiotics, and the patient was closely followed by the orthopedics service as an outpatient. At two-month follow-up, the patient continued to have mild swelling, stiffness, and decreased flexion range of motion of the affected PIP joint.

## DISCUSSION

There are no clear guidelines or consensus for the treatment and management of open dorsal PIP dislocations, including antibiotic use. A literature search revealed a paucity of current literature dedicated specifically to this type of injury; most were orthopedic review articles regarding closed PIP injuries with only a cursory mention of open dislocations. The most relevant papers were two case series from the 1980s^4^,^5^; however, these were reported from an orthopedic perspective and most of the patients were treated operatively.

Although open dorsal PIP dislocations may require open reduction, some are amenable to standard closed reduction, such as in our case. Preferably, anesthesia should be obtained via digital block after neurovascular assessment and prior to manipulation. As with closed dorsal dislocations, the PIP joint should be hyperextended (exaggerating the injury) while maintaining axial traction, applying volar-directed pressure on the middle phalanx, followed by gently flexing the PIP joint.^1^,^6^

**CPC-EM Capsule**

What do we already know about this clinical entity?

Open proximal interphalangeal (PIP) dislocations are often resistant to usual manual reduction techniques and may require surgical reduction.

What makes this presentation of disease reportable?

Open PIP dislocations are most commonly volar; dorsal dislocations can result in numerous complications.

What is the major learning point?

Open dorsal PIP dislocations may appear innocuous but are fraught with complications due to the potential for infection and damage to supportive structures.

How might this improve emergency medicine practice?

This case helps illustrate the need for specialist consultation and follow-up.

There are some differing recommendations in the orthopedic/sports medicine literature regarding splinting vs “buddy taping” the finger after successful reduction.^1^,^7^,^8^ However, most of these are in the context of closed dislocations with a stable PIP joint following reduction. A conservative and safe approach would be splinting at 20–30 degrees flexion^6^ and ensuring prompt follow-up with a hand specialist, since prolonged splinting is associated with increased stiffness and contractures and early range of motion is essential.^2^

Although the laceration may appear superficial and innocent, it represents a direct communication to the PIP joint and surrounding structures.^5^ If grossly contaminated, surgical washout is required.^9^ In our case, the wound was relatively clean and washout was accomplished in the ED with copious irrigation. Antibiotics were deferred per orthopedic recommendation since the wound was not grossly contaminated and had been thoroughly irrigated, and open reduction was not required. However, many authors do recommend empiric antibiotics in cases of open dislocations.^5^,^6^,^9^

## CONCLUSION

Open dorsal PIP dislocations may appear innocuous but are fraught with complications due to the potential for infection and damage to supportive structures.^5^ It is important for emergency providers to recognize the significance of an open injury and obtain appropriate specialty consultation. Patients should be educated regarding potential sequelae including infection, stiffness, swelling, pain, and contractures.^7^ Discharge instructions should also emphasize the importance of close follow-up with a hand specialist and compliance with rehab.
